# Experimental Chemotherapy for Chagas Disease: A Morphological, Biochemical, and Proteomic Overview of Potential *Trypanosoma cruzi* Targets of Amidines Derivatives and Naphthoquinones

**DOI:** 10.4061/2011/306928

**Published:** 2011-06-30

**Authors:** Solange L. de Castro, Denise G. J. Batista, Marcos M. Batista, Wanderson Batista, Anissa Daliry, Elen M. de Souza, Rubem F. S. Menna-Barreto, Gabriel M. Oliveira, Kelly Salomão, Cristiane F. Silva, Patricia B. Silva, Maria de Nazaré C. Soeiro

**Affiliations:** Laboratório de Biologia Celular, Instituto Oswaldo Cruz, Fundação Oswaldo Cruz, 21040-900 Rio de Janeiro, RJ, Brazil

## Abstract

Chagas disease (CD), caused by *Trypanosoma cruzi*, affects approximately eight million individuals in Latin America and is emerging in nonendemic areas due to the globalisation of immigration and nonvectorial transmission routes. Although CD represents an important public health problem, resulting in high morbidity and considerable mortality rates, few investments have been allocated towards developing novel anti-*T. cruzi* agents. The available therapy for CD is based on two nitro derivatives (benznidazole (Bz) and nifurtimox (Nf)) developed more than four decades ago. Both are far from ideal due to substantial secondary side effects, limited efficacy against different parasite isolates, long-term therapy, and their well-known poor activity in the late chronic phase. These drawbacks justify the urgent need to identify better drugs to treat chagasic patients. Although several classes of natural and synthetic compounds have been reported to act *in vitro* and *in vivo* on *T. cruzi*, since the introduction of Bz and Nf, only a few drugs, such as allopurinol and a few sterol inhibitors, have moved to clinical trials. This reflects, at least in part, the absence of well-established universal protocols to screen and compare drug activity. In addition, a large number of *in vitro* studies have been conducted using only epimastigotes and trypomastigotes instead of evaluating compounds' activities against intracellular amastigotes, which are the reproductive forms in the vertebrate host and are thus an important determinant in the selection and identification of effective compounds for further *in vivo* analysis. In addition, due to pharmacokinetics and absorption, distribution, metabolism, and excretion characteristics, several compounds that were promising *in vitro* have not been as effective as Nf or Bz in animal models of *T. cruzi* infection. In the last two decades, our team has collaborated with different medicinal chemistry groups to develop preclinical studies for CD and investigate the *in vitro* and *in vivo* efficacy, toxicity, selectivity, and parasite targets of different classes of natural and synthetic compounds. Some of these results will be briefly presented, focusing primarily on diamidines and related compounds and naphthoquinone derivatives that showed the most promising efficacy against *T. cruzi*.

## 1. Chagas Disease and Its Treatment

Chagas disease (CD) is caused by the intracellular obligatory parasite *Trypanosoma cruzi. * The life cycle of this parasite involves haematophagous triatomine insect vectors, diverse vertebrate mammalian hosts, and different developmental forms. Briefly, after bloodstream trypomastigotes are ingested by an insect, they are converted to epimastigotes, which proliferate and differentiate into metacyclic forms within the posterior intestine of the triatomine. These infective forms, released within the faeces and urine, can invade vertebrate cells and undergo another round of differentiation into the intracellular amastigote forms, which proliferate and then transform back to trypomastigotes, the form that disseminates the infection. CD is the major cause of infectious cardiopathy and represents an important public health problem; it is broadly dispersed in 18 developing countries in South and Central America [1]. It affects approximately eight million people in Latin America, of whom 30–40% either have or will develop cardiomyopathy, digestive megasyndromes, or both [2]. CD transmission occurs primarily via the vector (90%) but may also occur through blood transfusion and congenital transmission as well as laboratory accidents [3], organ transplantation [4], and ingestion of contaminated food and beverages [5]. Recently, CD has become a major concern due to globalisation, which results in immigration of infected individuals to nonendemic regions, thus spreading the disease [6]. Although both vectorial and transfusional transmission have sharply declined in the past 20 years due to Southern Cone countries' policies, several challenges still need to be overcome, including those related to sustainable public health initiatives, vector control strategies, and educational approaches [7, 8]. Thus, because epidemiological and transmission control characteristics of CD may vary according to each country's ecological conditions and adopted health policies, continuous epidemiological survey in conjunction with efficient and universal therapy of the infected individuals must be performed to maintain and even reduce the number of new acute cases [9].

CD affects mainly poor, rural and forgotten populations and has two consecutive clinical phases: the acute phase that appears shortly after infection, which ranges from flu-like symptoms to intense myocarditis (in approximately 10% of infected people), and the chronic symptomatic phase, which develops in approximately one-third of infected individuals after an asymptomatic period (indeterminate form) lasting years or decades [10, 11]. The main clinical manifestations of the chronic stage include cardiac and/or gastrointestinal involvement, and the variability in CD outcome has been related to host response and parasite heterogeneity [12]. Although its pathology is poorly understood, growing evidence has shown that parasitic persistence within the target organs associated with an unregulated host immune response is involved in pathogenesis, disease progression, and outcome [1, 13, 14]. 

Since its discovery by Chagas more than a hundred years ago [15], CD still poses many challenges, including its peculiar epidemiology, characterised by a variety of risk factors (diverse vectors and reservoirs and different forms of transmission and parasite isolates present in domiciliar, peridomiciliar, and sylvatic environments), and the lack of prophylactic therapies and effective chemotherapeutic schemes [10, 16, 17]. 

Nifurtimox ([3-methyl-4-(5*'*-nitrofurfurylideneamine) tetrahydro-4H-1,4-tiazine-1,1-dioxide], Nf) and benznida-zole (N-benzyl-2-nitroimidazole acetamide, Bz) were empirically introduced into the clinical therapy regime for CD over four decades ago. Neither drug is ideal because they present variable results depending on the phase of the disease (they are only effective in the acute and recent chronic phases of the infection), the dose and duration of the treatment, patient age, and endemic region in addition to showing undesirable secondary side effects [18, 19]. Additionally, differences in the susceptibility and natural resistance of different *T. cruzi* isolates to both nitroderivatives have also been reported [20]. It has been suggested that CD must be treated in all its stages, including acute (acquired or congenital), chronic reactivated (under immunosuppressive conditions), indeterminate, and early chronic phases, as determined by the presence of parasitic DNA using PCR analysis [21]. Additionally, although there is still no criterion of cure for symptomatic late chronic cases because most treated individuals show positive serology, recent data suggest the benefits of Bz therapy for chronic patients through the arrest of cardiac damage and a decrease in serology titres [22, 23]. These results reinforce a goal for identifying parasitic targets that is strengthened by the concept that chagasic cardiomyopathy is related to parasitic persistence within the target organs along with an unbalanced host immune response, which could be useful for a new CD therapy [24].

Since the introduction of Bz and Nf, only allopurinol and a few azoles, such as itraconazole, fluconazole, and ketoconazole, have moved to clinical trials [25–27]. In fact, drug development efforts for CD are almost exclusively in preclinical research, although phase II studies for the antifungal drug posaconazole and a prodrug of ravuconazole are being planned [28]. In addition, clinical data have demonstrated a positive effect of posaconazole in the therapy of a chronic chagasic patient with systemic lupus erythematosus [29]. However, the high costs of posaconazole may impair its use in CD. 

The gap between preclinical studies and clinical trials may be associated with the small amount of investments by pharmaceutical industries due to the low monetary return and to the previous mistaken concept that during the later stages of CD, parasitism is absent [30]. In addition, the lack of standardised protocols and the use of epimastigotes for drug screening may represent significant impairments for the discovery of novel anti-*T. cruzi* candidates [31]. Based on current knowledge regarding parasite and host physiology, a promising trypanocidal drug would include the following characteristics: (i) high activity against the parasitic forms present in mammalian hosts (intracellular amastigotes and bloodstream trypomastigotes), (ii) high activity against diverse *T. cruzi* strains for use in different endemic regions, (iii) efficacy against both acute and chronic infections, (iv) oral bioavailability in few doses, (v) low toxicity and improved safety with low potential for genotoxicity and teratogenicity given the potential use in children and women of reproductive age, (vi) low cost and high stability for a long shelf-life in tropical temperatures, (vii) high levels of tissue accumulation and long terminal half-lives, (viii) low risk for cardiotoxicity because the heart is the primary organ affected in chagasic patients, and (ix) low risk for interactions with hepatic cytochrome P450s to avoid drug-drug interactions because many patients use antiarrhythmic drugs and anticoagulants [28, 32].

## 2. Parasite Targets and Lead Compounds for New Drugs

Advances in proteomics, biochemistry, and in understanding the biological aspects of *T. cruzi* infection have allowed for the development of new approaches to identify parasite-specific targets and, thus, the design of novel potential drugs [33, 34]. It has been proposed that a rational therapy for *T. cruzi* should be directed against different parasitic metabolic targets, including ergosterol biosynthesis, trypanothione metabolism, cysteine protease, pyrophosphate metabolism, protein or purine synthesis, and DNA [21, 28, 35–37]. The combination of different drugs with the aim of achieving higher efficiency and lower toxicity is also an interesting therapeutic strategy [38]. Recent studies have demonstrated the successful synergism of posaconazole with amiodarone, an antiarrhythmic drug, against *T. cruzi in vitro* and *in vivo* [39]. Another relevant approach is the aetiological therapy of CD using carrier molecules, such as ruthenium complexes, that bind 14a-demethylase inhibitors [40] or benznidazole [41], improving both solubility and parasite specificity [28]. A recent study has shown the successful use of ruthenium complexes to deliver nitric oxide to *T. cruzi*-infected cells [42]. 

Proteomic approaches have been extensively applied for the evaluation of the expression, structure, and function of proteins on a large-scale, including their physiological role, expression regulation, and validity of genome annotations [43]. In trypanosomatids, the control of gene expression is particularly important because all protein-encoding genes are organised in large polycistronic transcription units, producing the RNA that will be processed by trans-splicing. Furthermore, the modulation of protein expression and, consequently, its function is directly related to posttranslational modifications in these protozoa [44, 45]. Proteomic studies have been performed to evaluate the mechanisms of *T. cruzi* resistance/susceptibility to drugs [44–46]. 

In the present review, we summarise *in vitro* and *in vivo* results on the efficacy, toxicity, selectivity, and cellular targets of aromatic diamidines and naphthoquinone derivatives, two groups of compounds with promising efficacy against *T. cruzi*. In this framework, morphological techniques, such as light (confocal and fluorescence) and electron (transmission and scanning) microscopy, have been employed. Other cellular (flow cytometry), biochemical (respirometry and fluorimetry), and proteomic (bidimensional electrophoresis and mass spectrometry) approaches have also been employed to identify specific targets in the parasite.

### 2.1. Aromatic Diamidines and DNA Damages

Diamidines, such as pentamidine, propamidine, and diminazene aceturate, have been successfully used in human and veterinary medicine, and they are the first class of drug extensively employed for early-stage human African trypanosomiasis and for cutaneous leishmaniasis caused by *Leishmania guyanensis* [47–49]. Their major drawbacks are poor oral bioavailability and severe side effects. To overcome these issues, new dicationic analogues and prodrugs have been synthesised by different medicinal chemistry groups and widely assayed *in vitro* and *in vivo* [50]. One of the most promising compounds is an orally effective prodrug of furamidine (DB75) named DB289, which has been in phase III clinical trials for African trypanosomiasis [51]. Unfortunately, recent results at an extended dosage led to the withdrawal of DB289 from human trials due to toxicity issues.

Despite the strong activity of these dicationic compounds against African trypanosomes, few have been assayed as anti-*T. cruzi* candidates [50]. Our team has recently been working on the potential effect of diamidines and congeners against this parasite using both *in vitro* and in vivo models to compare analogues with different structures, cationic centres, and effective motifs [19]. Our data have clearly shown the promising activity of some of these compounds, which displayed high therapeutic windows [52–56].

Although DB75 and its N-phenyl-substituted analogue (DB569) display equivalent DNA-binding properties, DB569 exhibited higher *in vitro* activity against different strains and stages of *T. cruzi*, with IC_50_ values in the low micromolar range [57]. Due to the characteristic fluorescence of these diamidines, their localisation in DNA-enriched organelles was determined due to strong labelling of the kDNA [58]. Flow cytometry and transmission electron microscopy (TEM) analysis also demonstrated that DB75 and DB569 disturb parasite mitochondria and nuclei, leading to morphological characteristics of programmed cell death, such as higher levels of apoptotic-like parasites observed after the treatment with DB569 [57, 59]. These findings stimulated further *in vivo* analysis with this analogue, which showed a reduction in the number of parasites and CD8^+^ T cells in heart tissues and reversion of electrocardiogram (ECG) alterations in acutely *T. cruzi*-infected mice, leading to an increase in the survival rates [60]. The ECG protection provided by DB569 was also found during the chronic infection of experimental animals, suggesting that the reversion observed in treated animals may be associated with the reduction in cardiac CD8^+^ lymphocyte infiltration and parasitism, ultimately contributing to their survival [59, 60].

A diarylthiophene diamidine (DB1362) was evaluated against amastigotes and bloodstream trypomastigotes of *T. cruzi* and showed good efficacy *in vitro* at submicromolar concentrations, inducing low host cytotoxicity. This diamidine presented a dose-dependent trypanocidal effect after incubation in the presence of plasma constituents (mouse blood), exhibiting IC_50_ values similar to those found in the absence of blood, pointing to its potential prophylactic application in blood banks. TEM and flow cytometry have shown that in bloodstream parasites the most important alterations were in kinetoplast organisation and mitochondrial membrane potential [54]. In an acute *T. cruzi* experimental mouse model, treatment with two doses of 25 mg/kg DB1362 (at the onset and at the parasitaemia peak) led to a 40% decrease in the circulating trypomastigotes and cardiac parasitism (similar levels to Bz) and protected against ECG alterations, leading to a 100% survival rate [54].

Studies on the biological and ultrastructural effect and subcellular localisation of six novel diamidines in *T. cruzi* confirmed their low toxicity towards mammalian cells (LC_50_ > 96 *μ*M) and demonstrated that small linear molecules (DB1627, DB1646, and DB1670) were not effective. However, the other three diamidines (DB1645, DB1582, and DB1651) were active, with IC_50_ values between 0.15 and 13.3 *μ*M against bloodstream and intracellular amastigotes [61]. 

Several potential transporters of diamidines have been described in other parasites, such as African trypanosomes, *Leishmania* species and *Plasmodium falciparum* [62–64]. However, the mechanism of diamidine uptake in *T. cruzi* is unknown and requires further investigation. The intrinsic fluorescence of some of these compounds allows for monitoring their localisation, as previously reported in studies with African trypanosomes [50, 65, 66]. Some of these diamidines, like DB1582 and DB1651, were localised in parasitic nuclei and kDNA (with higher intensity in kDNA) and within punctate non-DNA-containing cytoplasmic organelles usually localised in the anterior portion of trypomastigotes and near the nuclei and kinetoplasts in amastigotes, which are possibly acidocalcisomes, as previously described for *T. brucei* [65]. As previously suggested for African trypanosomes, the localisation of these compounds within these acidic organelles could play a role in their mechanism of action and/or act as storage sites [65, 66], but additional studies are needed to clarify this matter. Batista et al. [61] demonstrated that these diamidines caused striking alterations in the mitochondria and kinetoplasts of *T. cruzi*, and some of them also induced disorganisation of microtubules, with the appearance of multiple axoneme structures in trypomastigotes [52, 61]. No major alterations have been reported in either subpellicular or flagellar microtubules of *T. cruzi* treated with drugs that target microtubules, such as taxol, colchicines, and vinblastine, possibly due to the high content of acetylated tubulin and/or poly-glutamylation of tubulin in these parasites [67]. Because these structures are more resistant to microtubule disrupters in trypanosomatids compared to mammalian cells, they may represent interesting targets for drug development and justify further investigations.

Although the exact mechanism of action of diamidines on *T. cruzi* and other trypanosomatids has not been clearly demonstrated, it is likely that multiple modes of action may be responsible and that compound uptake represents a fundamental step in their action and selectivity [50, 68]. One of the long-hypothesised mechanisms of diamidines is related to their selective binding to sequences rich in adenosine and thymine (AT) of kDNA minicircles, leading to kinetoplast destruction and parasite death [58, 69]. Because the kDNA of trypanosomatids contains high numbers of AT-binding sites in thousands of repeated minicircles, it is possible that these structures represent potential specific targets for diamidines [50]. However, although these compounds are excellent minor groove DNA-binders, this interaction itself cannot fully explain their biological activity. Recent reports have suggested that their association with DNA could represent an initial step followed by topological changes leading to molecular instability and destruction and/or modification of DNA-protein interactions, leading to replication errors, DNA degradation, and parasite death [70]. TEM studies have shown that the organisation of mitochondria and kinetoplasts in *T. cruzi* is highly altered by several diamidines and related compounds, such as arylimidamides (AIAs), at concentrations that do not affect mammalian host cells [19, 51, 58]. AIAs, previously known as reversed amidines, have extraordinary activity against both *Leishmania* [71–73] and *T. cruzi* [52, 53, 55, 56, 74]. They differ from other furan analogues because the amidine is bound to the central aromatic linker via a nitrogen atom rather than a carbon atom [72]. 

Flow cytometry data have confirmed that diamidines and AIAs target the mitochondria-kinetoplast complex of *T. cruzi* through interference with the mitochondrial membrane potential [53, 54]. *In vitro* screening of novel diamidines against *T. cruzi* has shown that these compounds localise to a higher extent within the kinetoplast than in the nucleus, and no correlation was found between trypanocidal activity and higher kDNA accumulation [75, 76], as previously reported in *T. brucei* [65]. Other targets for diamidines that also have been proposed include the inhibition of tyrosyl-DNA phosphodiesterase, topoisomerases, protein kinase A, proteases, and polymerases [51, 77, 78].

To better understand the mechanism of action of these aromatic compounds, a study of the possible correlation between kDNA-binding properties of 13 amidines with their trypanocidal efficacy against *T. cruzi* was performed. Four diamidines (DB75, DB569, DB1345, and DB829), eight arylimidamides (DB766, DB749, DB889, DB709, DB613A, DB1831, DB1852, and DB2002), and one guanylhydrazone (DB1080) were assayed using thermal denaturation (*T*_*m*_) and circular dichroism (CD) studies using both whole *T. cruzi* purified kDNA and a conserved synthetic parasitic sequence corresponding to the biological activity of each compound [79]. The findings suggest that the strong interaction of amidines with kDNA may not be sufficient to generate and trigger their trypanocidal activity, and other mechanisms of action may be involved and/or associated. 

AIAs have potent *in vitro* dose-dependent activity against *T. cruzi*, showing superior trypanocidal activity compared to diguanidino cationic groups and other classical diamidines [52, 56]. Recently, a monoamidine, an arylimidamide, and a guanylhydrazone were evaluated, and the data showed that all compounds exerted, at low micromolar doses, a trypanocidal effect upon both intracellular and bloodstream parasites [74]. However, the potency and selectivity of DB613A, an AIA, towards intracellular parasites (with a selective index >126) corroborated previous results that demonstrated the high promising trypanocidal activity of these compounds. 

*In vitro* and *in vivo* studies conducted with a novel AIA, DB766, showed its strong trypanocidal activity and excellent selectivity for intracellular amastigotes and trypomastigotes (Y strain), the two relevant parasite forms present in mammalian hosts, exhibiting IC_50_ values of 25 and 60 nM, respectively [61]. DB766 also exerted striking effects on a wide panel of different parasite strains, including those naturally resistant to Nf and Bz, displaying higher activity *in vitro* than Bz and gentian violet, which are important requirements for identifying a potential anti-*T. cruzi* agent. It is also important to point that DB766 was active against parasite isolates that circulate in peridomiciliar and sylvatic ecotopes from two different regions in Brazil: (i) the northeast (Jaguaribe Valley, Ceará state) that represents an important area for CD surveillance, where high rates of natural triatomine infection are observed (mostly *T. cruzi* type I lineage) and vectorial control still requires effort to avoid new cases of human transmission, and (ii) the Amazon region that presents an important new epidemiologic challenge due to the increasing reports of human acute cases, mainly by oral contamination as well as by wild triatomine vectorial transmission [61]. Next, as no major acute toxicity was noted with uninfected mice, this AIA was moved to models of acute and chronic experimental *T. cruzi* infection. DB766 effectively reduced the parasite load in blood and cardiac tissue and presented similar efficacy to Bz in mouse models of acute and chronic *T. cruzi* infection (using Y and Colombiana strains, which are considered modered and highly resistant to Bz), using few oral and intraperitoneal doses up to 100 mg/kg/day given after the establishment of parasite infection. As *T. cruzi* is an obligatory intracellular parasite and reservoirs of amastigotes can be found in quite distinct organs and tissues, the ability of AIAs (including DB766) to traverse host cell membranes possibly by passive diffusion and or transporters associated with their extensive tissue binding in liver, spleen, and heart [80] makes this class of compounds very attractive for CD treatment. In fact, the pharmacokinetic properties of DB766 are especially relevant since the poor activity of the nitroheterocyclic compounds during the chronic stages of CD may be related to short half-lives and limited tissue penetration of Bz and Nf. The efficacy of DB766 upon several strains *in vitro* and *in vivo* is a very important finding since this parasite comprises numerous clonal populations with distinct characteristics such as different sensitivity to Nf and Bz, diverse biological parameters and enzymatic diversity, and strain heterogeneity may also be related to the different clinical manifestations and outcomes in CD. Thus, the broad spectrum of DB766 activity is a desirable characteristic of a novel compound for the treatment of this neglected illness. In acute experimental models of *T. cruzi* infection, DB766 ameliorated heart alterations, reduced hepatic and heart lesions induced by the infection, and provided 90–100% protection against mortality. DB766 also presented high *in vivo* efficacy when given orally at 100 mg/kg/day, showing similar effect to the Bz-treated group [61]. Interestingly, the oral administration of DB766 (at 100 mg/kg/day) leads to reduced circulating and cardiac parasitism besides protecting against mortality without causing major side effects. These results suggest that although not being a prodrug, sufficient quantities of DB766 were absorbed from the mouse gastrointestinal tract, effectively delivering this AIA across the gut mucosa, similar to that reported for the AD prodrug DB289. The bulk of these results demonstrate the promising trypanocidal efficacy of DB766, suggesting that AIA may represent a new lead candidate for CD treatment. Interestingly, DB766 produced a clear dose-dependent decrease in parasitaemia in the liver, spleen and bone marrow in two experimental models of *L. donovani* infection [80]. Additionally, pharmacokinetics, mutagenicity, and toxicity studies revealed that this AIA did not exhibit mutagenicity (AMES test), displayed low acute toxicity, had moderate oral bioavailability, was distributed to different tissues (such as the liver and spleen), presented large areas of distribution, and showed an elimination half-life ranging from one to two days in mice [80]. 

Six novel aromatic amidinic compounds were tested *in vitro* to determine activity against the infective and intracellular stages of *T. cruzi* and evaluate their selectivity and toxicity towards primary cultures of cardiomyocytes [56]. The data demonstrated that all of the aromatic amidines were active against *T. cruzi in vitro* and that the arylimidamide DB1470 was the most effective compound, presenting IC_50_ values at submicromolar levels and a good selectivity index and maintaining significant trypanocidal activity at 4°C in the presence of blood constituents [56]. Interestingly, AIAs, such as DB1470 and DB766, exhibited potent trypanocidal activity against *T. cruzi* in the presence of blood constituents [52, 55]. This characteristic is highly desirable for new potential trypanocidal agents for use in blood banks in endemic areas. Unfortunately, although transfusional control has led to a decline in the number of new blood bank-related infections, it is not universally performed. The only trypanocidal agent available for chemical prophylaxis of blood in areas of high endemicity is gentian violet, which is a toxic cationic dye that gives a purple colour to the blood and stains the skin and mucosa of the recipients [10, 81]. These limitations encourage the search for new compounds that could be used in blood bank prophylaxis; thus, AIAs such as DB766 represent promising agents for further evaluation for this purpose [55]. In summary, the efficacy of diamidines and congeners, like AIAs, against *T. cruzi* requires further studies to help establish a valuable scheme of therapy for CD.

### 2.2. Naphthoquinone Derivatives and Mitochondrial Dysfunction

Naphthoquinones are compounds present in different families of plants that serve as vital links in the electron transport chains in the metabolic pathway and participate in multiple biological oxidative processes [82, 83]. They are considered privileged structures in medicinal chemistry due to their biological activities and structural properties [84]. The redox cycling of quinones may be initiated by either a one- or two-electron reduction. The one electron reduction leads to the formation of semiquinones, unstable intermediates that react rapidly with molecular oxygen, generating free radicals. All of these highly reactive oxygen species (ROS) may react directly with DNA or other cellular macromolecules, such as lipids and proteins, leading to cell damage [85]. This reaction results in shunting electrons toward oxygen, an ineffective pathway for reduction equivalents otherwise used for cytochrome P450 reductase-dependent reactions [86–89]. Another alternative is reduction by two electrons, leading to the formation of hydroquinone, mediated by DT-diphorase [90, 91]. This enzyme reduces toxic, reactive, and unstable quinones, bypassing the creation of toxic intermediates (e.g., a semiquinone radical), and sparing the cell from ROS formation. Whether the two-electron reduction of a quinone leads to detoxification or to activation of oxidative stress depends upon the rate of autoxidation of the formed hydroquinone [86]. If this rate is low under physiological conditions, conjugation may occur before oxidation. As a consequence, the two-electron reduction will lead to detoxification, and an increase in the DT-diphorase activity in tissues would be expected to decrease the toxicity of the quinone. If, however, the hydroquinone is rapidly oxidised, only a minor fraction may be conjugated before oxidation occurs, and hydroquinone formation would constitute an activation reaction. As a result, enhanced tissue levels of NQO1 would be expected to increase the toxicity of the quinone [92]. Quinones are oxidants and electrophiles, and the relative contribution of these properties to both their toxic and therapeutic activities is influenced by their chemical structure [93]. Two major mechanisms of quinone cytotoxicity have been proposed: stimulation of oxidative stress and alkylation of cellular nucleophiles, which are the mechanisms of action of encompass a large range of biomolecules [94]. Cellular damage can also occur through the alkylation of crucial proteins and nucleic acids.

In addition to their widespread presence in nature, the great interest in the study and mechanisms of action of compounds with a quinoidal structure is due to their multiple roles in organisms. Several quinonoids isolated from traditional medicinal plants are being investigated for their anticancer properties [95]. The antiprotozoal activities of naphthoquinones have been reported, and several of them have been identified as possible leads for drug development [96–98].

Lapachol is easily isolated from the heartwood of trees of the Bignoniaceae family abundant in tropical rainforests, while both *α*-lapachone and *β*-lapachone are present only in small amounts. In Brazil, more than 46 types of such woods, popularly known by the name “ipes” (*Tabebuia* sp.), have been described. In folk medicine, especially among Indian populations, plants containing naphthoquinones have been employed for the treatment of different diseases, such as cancer [99, 100]. The inner bark of *Tabebuia avellanedae*, commonly known as “pau d'arco” (lapacho, taheebo), is used as an analgesic, an anti-inflammatory, an antineoplastic, and a diuretic by the local people in the northeastern regions of Brazil [101]. 

Previous reports have shown that against *T. cruzi* epimastigotes, *β*-lapachone increases the generation of reactive oxygen species through formation of the semiquinone radical, leading to lipid peroxidation and inhibition of nucleic acid and protein synthesis [102–106]. *T. cruzi* is known to be deficient in reactive oxygen and nitrogen species detoxification and for being especially sensitive to oxidative stress conditions [107]. Its single mitochondrion, containing a branched electron transport chain and a specialised kDNA region [77], is an extraordinary drug target [33]. The ultrastructural injuries observed in *β*-lapachone-treated epimastigotes [108] together with the increase in the generation of hydrogen peroxide clearly demonstrates the mitochondrial susceptibility of *T. cruzi* to naphthoquinones. Unfortunately, no trypanocidal effect was observed in suspensions containing foetal calf serum or rabbit haemoglobin solution, suggesting that *β*-lapachone could be inactivated by either reduction in the presence of oxyhaemoglobin or interaction with serum proteins [109].

Due to the easy access to natural sources of quinones from Brazilian flora and the synthetic mechanisms developed by the group of Dr. Pinto (NPPN/UFRJ) exploring the electrophilicity of 1,2-quinoidal carbonyls [110–113], naphthoquinones have been used as starting points for medicinal chemistry studies. Since the 90s, our group has been studying the anti-*T. cruzi* activity of this class of chemicals [114]. An initial screening was performed on 60 derivatives obtained through the reaction of several naphthoquinones with common reagents from heterocyclic chemistry, leading to 14 oxazolic, 30 imidazolic, and 10 other related heterocyclic compounds [115–119]. Comparing the activity of the original naphthoquinones and their derivatives, we concluded that minor structural features involved with an increase in lipophilicity, such as the furane moiety, the presence of a methoxyl group, and an aliphatic side chain, led to an increase in the effect on *T. cruzi*. It is possible that a lipophilic character allows better penetration of the compound through the plasma membrane of the parasite. The activity of the synthesised compounds on *T. cruzi* showed no uniform behaviour and was in some cases higher, lower, or similar to the activity of the original naphthoquinones from which they were obtained. For the naphthooxazoles assayed, there was no correlation between biological activity and the type of the mono-oxygenated ring (pyrane *versus* furane). As shown for naphthoquinones, a lipophilic characteristic, introduced by this appendage, and the presence of a methoxyl or a phenyl group increased the trypanocidal activity. A characteristic of the synthesised naphthoimidazoles was that most of them had aromatic groups containing electron-releasing or electron-withdrawing groups attached to the imidazole ring, and the most active compounds against *T. cruzi* were obtained from *β*-lapachone (see Section 2.3) [115, 119]. 

Another group of naphthoquinone derivatives was also synthesised and assayed on trypomastigote forms, including *β*-lapachone- and nor-*β*-lapachone-based 1,2,3-triazoles and 3-arylamino-nor-*β*-lapachones [120, 121]. 1,2,3-Triazoles are an important class of heterocyclic compounds due to their wide range of biological activities. The strategy of molecular hybridisation linking them to naphthoquinones resulted in compounds endowed with redox properties and a trypanocidal profile. The 1,2,3-triazole derivatives of nor-*β*-lapachone were more active than the original quinone, and the apolar phenyl-substituted derivative (2,2-dimethyl-3-(4-phenyl-1,2,3-triazol-1-yl)-2,3-dihydro-naphtho[1,2-*b*]furan-4,5-dione) was the most active compound (IC_50_/24 h = 17.3 ± 2.0 *μ*M) [120]. Such activity could be due to its higher lipophilic character, which allows better penetration through the parasite's plasma membrane. In addition, the key intermediate azides used for the synthesis of both the *β*-lapachone and nor-*β*-lapachone series of 1,2,3-triazoles displayed higher activity than Bz (IC_50_/24 h = 103.6 ± 0.6 *μ*M). In the case of nor-*β*-lapachones-3-arylamino-substituted compounds, the insertion of chlorine, bromine, nitro, and methoxy groups into the arylamino ring intensified the trypanocidal activity. 

Using nor-lapachol as a starting point, substituted *ortho*-naphthofuranquinones, a nonsubstituted *para*-naphthofuranquinone, an oxyrane and an azide were prepared. Using *α*-lapachone as a base, a new nonsubstituted *para*-naphthofuranquinone was prepared. The most active compounds were three *ortho-*naphthofuranquinones with trypanocidal activity higher than that of Bz [122]. In another set of experiments, three new naphthofuranquinones were synthesised and assayed on *T. cruzi*. Two of them were obtained by the addition of iodine to C-allyl-lawsone (2-hydroxy-3-allyl-naphthoquinone) followed by cyclisation, generating a furan ring; the third was obtained through an acid-catalysed reaction by dissolution of the original quinone in sulphuric acid. These compounds were active on bloodstream trypomastigote and epimastigote forms with IC_50_ values between 165–640 and 2.5–25 *μ*M, respectively [123]. The treatment of infected murine macrophages caused a dose-dependent decrease in the percentage of infection with low toxicity to host mammalian cells (over 100 *μ*M), with IC_50_/24–96 h values for intracellular amastigotes between 1.2 and 3.5 *μ*M [124]. An ultrastructural analysis of treated epimastigotes and trypomastigotes indicated a potent effect of the three naphthofuranquinones on parasitic mitochondria, which appeared drastically swollen and with a washed-out matrix profile. Fluorescence-activated cell sorting analysis of rhodamine-123-stained *T. cruzi* showed that these naphthofuranquinones caused a potent dose-dependent collapse of the mitochondrial membrane potential (ΔΨ_*m*_), especially in epimastigote forms. Such a collapse represented a 30–60% decrease in the parasitic ΔΨ_*m*_. These compounds also specifically decreased mitochondrial complex I-III activities parallel to a reduction in succinate-induced oxygen consumption between 64–75% in epimastigotes and 72–92% in trypomastigotes. Mitochondrial hydrogen peroxide formation was also increased 1.3–4.5-fold in epimastigotes after treatment with naphthofuranquinones. Our results indicated that the trypanocidal action of these quinones was associated with mitochondrial dysfunction, leading to increased reactive oxygen species generation and parasitic death [124]. The easy synthetic route of these compounds in the laboratory opens the possibility of large-scale production with high yields for assays in experimental mouse models.

### 2.3. Naphthoimidazoles and Putative Targets

Among the different classes of naphthoquinone derivatives screened against *T. cruzi* (see Section 2.2), the most active derivatives against bloodstream trypomastigotes were three naphthoimidazoles derived from *β*-lapachone with the aromatic moieties phenyl (N1), 3-indolyl (N2), and methyl-*p*-phenyl (N3), which were selected for further studies [84, 85]. They were also active against intracellular amastigotes and epimastigotes (Table 1) and showed toxicity to the host cell in concentrations greater than 100 *μ*M. The most susceptible form of the parasite was the intracellular amastigotes, with an IC_50_/24 h between 6.5 and 9.0 *μ*M [125, 126]. The highest activity against bloodstream forms was observed for N2, with IC_50_/24 h values of 12.3 ± 1.2 and 61.6 ± 3.6 *μ*M for 0% and 100% blood, respectively. In epimastigotes, N3 was the most effective, with IC_50_/24 h values of 30.7 ± 3.6 *μ*M. All three compounds also blocked the cell cycle (up to 96% inhibition of DNA duplication), inhibited succinate cytochrome c reductase (16–42%) and metacyclogenesis (IC_50_/96 h values between 0.35–0.66 *μ*M) and induced extensive morphological damage to the mitochondria, Golgi complex and reservosomes. In treated trypomastigotes, an altered kinetoplast network, mitochondrial swelling, plasma membrane blebbing and DNA fragmentation were found [125, 126]. DNA fragmentation was also evaluated using total DNA electrophoresis and flow cytometry techniques and showed a maximum of 75% TUNEL+ trypomastigotes after treatment with the naphthoimidazoles.

An investigation into their mode of action led to the characterisation of mitochondria, reservosomes, and DNA as their main targets and stimulated further studies about death pathways. Ultrastructural analysis revealed both autophagic (autophagosomes) and apoptotic-like (membrane blebbing) phenotypes. In epimastigotes and trypomastigotes, the naphthoimidazoles induced the formation of concentric membranes, autophagosomes with a loss of matrix electron density, and endoplasmic reticulum profiles surrounding different structures [127]. Apoptosis-like features, such as the release of mitochondrial cytochrome c to the cytosol, has also been detected in N3-treated parasites. Flow cytometry analysis showed a small increase in phosphatidylserine exposure in N2-treated trypomastigotes and a large increase in the percentage of necrosis caused by N1 and N2. These death phenotypes were not detected in treated epimastigotes. A strong increase in the labelling of monodansyl cadaverine (a well-known autophagic marker) was observed up to 45 and 71% in treated trypomastigotes and epimastigotes, respectively. The inhibition of the trypanocidal action of the three naphthoimidazoles by wortmannin or 3-methyladenine together with the overexpression of *ATG3, ATG4, ATG7,* and *ATG8* genes in treated epimastigotes and the ultrastructural evidence pointed to autophagy as the predominant phenotype induced by these compounds [127, 128]. 

To assess the mechanism of action of the naphthoimidazoles N1, N2, and N3, treated epimastigotes were submitted to two-dimensional gel electrophoresis (2-DE) and mass spectrometry to monitor changes in the protein patterns of different important pathways in *T. cruzi*. Results showed that 9 of the 30 proteins altered were mitochondrial, reinforcing previous morphological and biochemical studies that showed this organelle as the main target of these drugs [125, 126]. Treatment with the compounds led to an upregulation of three proteins: heat-shock protein 85, enolase 1 and trypanothione synthetase (Table 2). The overmodulation of the trypanothione pathway strongly suggests that increased scavenging is necessary due to the increased thiol content in treated parasites, as previously described for trypanosomatids [129].

The downregulation of 26 proteins was observed: 7 in N1, 14 in N2, and 15 in N3. Table 2 summarises all modulated proteins after treatment. A strong decrease (20–80%) in protein levels was detected after treatment with the three naphthoimidazoles. Energy metabolism is an important target of these compounds. The expression of enzymes, such as enolase, pyruvate dehydrogenase, and cytochrome c oxidase, were altered in treated epimastigotes. Interference with ATP generation occurred in different stages of the pathway, including glycolysis, the citric acid cycle, and the mitochondrial electron transport chain. The main structural target of naphthoimidazoles was microtubules. A remarkable decrease in the *α*- and *β*-tubulin content was detected in epimastigotes treated with the three compounds. Interestingly, previous ultrastructural data showed no damage to subpellicular and flagellar conformations after treatment [125, 126]. ELISA showed a decrease in tyrosinated tubulin content, suggesting interference with intracellular vesicle traffic and/or mitotic spindle formation, data reinforced by the blockage of mitosis in naphthoimidazole-treated epimastigotes [126, 130]. Amino acid metabolism was one of the most important targets of the naphthoimidazoles. The levels of tyrosine aminotransferase, elongation factors and different heat-shock proteins were affected by N1, N2, and N3, leading to a decrease in the amino acid synthesis and, consequently, an imbalance in proteins important for cell survival. N2 and N3 also arrested the latter steps of sterol biosynthesis through the downregulation of sterol 24-C-methyltransferase (Table 2). The partial impairment of this pathway could lead to alterations in the lipid composition, with a loss of membrane fluidity. Proteomic and ultrastructural data showed no evidence of necrosis or apoptosis-like cell death in treated parasites [127, 130]. A further detailed study on metabolic interactions is crucial to further elucidate the mechanisms through which naphthoimidazoles act.

## 3. Concluding Remarks

Despite the drawbacks found during the therapy of chagasic patients with Bz and Nf, in the last decades, only a few compounds have moved to clinical trials, possibly due to the low investments allocated to this area and the lack of standardised protocols for drug screening [31]. As previously noted, “the more leads/approaches that progress to investigational drug candidates, the better chance that new treatments for this often fatal infection will be available to patients in the near future” [131]. Thus, the recent implementation of high-throughput compound screening against *T. cruzi* will allow for the rapid evaluation of several thousands of compounds per month against intracellular amastigotes, which represent an important tool that may yield the identification of novel promising compounds that can move to clinical trials [28]. 

However, these promising trypanocidal candidates will need more complete pharmacological and safety test analyses to be considered for clinical trials. However, consistent care must be provided through the adoption of a biopsychosocial model, considering patient therapy in the context of biological, psychological, and social factors, and economic difficulties, which can compromise quality of life [132, 133]. Finally, another important limitation related to moving new compounds towards clinical trials is the absence of feasible markers to monitor the progression of the chronic disease and affordable, efficient, and accessible diagnostic tests. Thus, the development of new drugs for most parasitic diseases requires a multidisciplinary approach involving diverse research areas, such as molecular and cellular biology, chemistry and biochemistry, pharmacology, and toxicology, to provide new insights related to the development and discovery of more selective compounds that could be used for Chagas disease therapy.

As concluded by Abad-Franch et al. [8], improved specific chemotherapy, including more practical formulations (e.g., paediatric) or combinations of existing drugs, and a better understanding of pathogenesis as well as the relative contribution of the parasite and host genetic makeup are clearly needed. New strategies for drug design have been improved by the recent results in *T. cruzi* biochemistry, allowing for better elucidation of the effects of trypanocidal agents. 

## Figures and Tables

**Table 1:** IC_50_ values/1 d (*μ*M) for the effect of N1, N2, and N3 against *T. cruzi* ^a^.

	Trypomastigotes		Epimastigotes	Amastigotes	
	0% blood	100% blood		Extracellular^b^	Intracellular^c^
N1	35.8 ± 1.2^d^	62.1 ± 3.0	82.8 ± 7.4	13.4 ± 1.1	9.0 ± 2.9
N2	12.3 ± 1.2	61.6 ± 3.6	36.0 ± 1.9	12.4 ± 1.9	6.5 ± 1.0
N3	28.2 ± 0.9	68.3 ± 7.3	30.7 ± 3.6	9.7 ± 0.2	7.2 ± 0.2

^a^References [123, 124].

^b^Tissue-cultured derived amastigotes.

^c^Number of amastigotes/100 peritoneal macrophages.

^d^Mean ± SD of at least 3 independent experiments.

**Table 2:** Modulated proteins in naphthoimidazoles-treated epimastigotes^a^.

Protein description	Expression status	Treatment
Trypanothione synthetase	upregulated	N1, N2, N3
Mitochondrial heat shock	downregulated	N1
Heat shock protein 60	downregulated	N2
Elogation factor 1-*α*	downregulated	N1
Enolase 1	upregulated	N3
Glutamamyl carboxypeptidase	downregulated	N1
Heat shock protein 85	upregulated/downregulated	N1, N2, N3
Tyrosine aminotransferase	downregulated	N3
Cytochrome C oxidase subunit IV	downregulated	N2
Activated PKC receptor	downregulated	N3
Hypothetical protein	downregulated	N2
*β*-tubulin	downregulated	N1, N2
Pyruvate dehydrogenase E1 *β* subunit	downregulated	N3
Sterol 24-C-methyltransferases	downregulated	N2, N3
Elongation factor 1-*β*	downregulated	N2
Proteosome *α* 7 subunit	downregulated	N1
Peroxiredoxin	downregulated	N2
Hypothetical protein	downregulated	N1
IgE-dependent histamine-releasing factor	downregulated	N3
Elongation factor 2	downregulated	N2
Dehydrogenase	downregulated	N3
Cystathionine *β*-synthase 6	downregulated	N2
*α*-tubulin	downregulated	N3

^a^Reference [128].
